# Correction to “The Importance of Verifying the Novelty of a Finding and the Value of Combining Results”

**DOI:** 10.1002/acn3.70201

**Published:** 2025-09-18

C. Jimenez‐Mallebrera, “The Importance of Verifying the Novelty of a Finding and the Value of Combining Results,” *Annals of Clinical and Translational Neurology* 9, no. 6 (2022): 893–894, https://doi.org/10.1002/acn3.51572.

The authors would like to acknowledge that the following funding information was inadvertently omitted in the original publication.

*This work was supported by the Instituto de Salud Carlos III (grant reference PI19/0122) and co‐funded by the European Union*.

We apologize for this error.

